# 

**Published:** 1910-06

**Authors:** 


### CONTENTS.

ORIGINAL COMMUNICATIONS—

Practical Aids in Medical and Surgical Diagnosis. By Lewis M. Gaines, A. B., M. D., Atlanta, Ga. .... 109
Further Experience With Vaccine in Tuberculosis. By Dr. E. C. Thrash, Atlanta, Ga. .... 116
Treatment of Chronic Prostatitis. By Alfred L. Fowler, M. D., Atlanta, Ga. .... 120
The Doctor, The Public and Medical Legislation. By Dr. J. G. Dean, Dawson, Ga. 133
Brain Tumor With Exhibit of Two Living Cases. By Wesley Taylor, B. S., M. D., Atlanta, Ga. .... 137
Alcoholism and Drug Addictions. By W. A. Starnes, M. D., Atlanta, Ga. .... 141
Hook Worm Eradication. By L. J. Sharp, Commerce, Ga. .... 146
Purpura. By L. C. Allen, M. D., Hoschton, Ga. .... 150

EDITORIALS .... 155
NEWS AND NOTES .... 156
BOOK REVIEWS .... 165

